# Comparative associative and discriminative value of inflammatory, coagulation, hypoxia–perfusion, and electrolyte–metabolic indices for early respiratory physiological decompensation in acute pancreatitis

**DOI:** 10.3389/fphys.2026.1775368

**Published:** 2026-04-22

**Authors:** Xiangyin Lv, Jiejun Hu, Jiao Bao

**Affiliations:** 1 Department of Gastroenterology, Affiliated Dongyang Hospital, Wenzhou Medical University, Dongyang, Zhejiang, China; 2 Department of Emergency, Affiliated Dongyang Hospital, Wenzhou Medical University, Dongyang, Zhejiang, China

**Keywords:** acute pancreatitis (AP), composite index (CI), electrolyte-metabolic disturbance, respiratory failure (RF), risk stratification

## Abstract

**Background:**

Respiratory failure is a significant determinant of early mortality in cases of acute pancreatitis; however, the relative contributions of various pathophysiological processes remain ambiguous. Comparing the associative and discriminative values of inflammatory, coagulation, hypoxia-perfusion, and electrolyte-metabolic indices for respiratory failure holds substantial clinical implications.

**Methods:**

We conducted a retrospective cohort study involving adult patients hospitalized with acute pancreatitis from 2019 to 2023. Four composite indices, which reflect systemic inflammation, coagulation activation, hypoxia-perfusion impairment, and electrolyte-metabolic disturbance, were constructed from routine laboratory measurements obtained within 24 hours of admission. The primary outcome was defined as respiratory failure occurring within 3 to 7 days of hospital admission, or respiratory−related death. Associations, dose-response relationships, and discriminative performance were evaluated using multivariable logistic regression, restricted cubic splines, and receiver operating characteristic analyses.

**Results:**

Among 438 eligible patients, 54 (12.33%) developed respiratory failure or died within 3 to 7 days of admission. All four indices were associated with the primary outcome in univariable analyses. After mutual adjustment, electrolyte-metabolic disturbance emerged as the only dimension independently associated with the primary outcome, with an adjusted odds ratio of 1.32 per 1-SD increase (95% CI 1.16-1.51). This index demonstrated a near-linear dose-response relationship and outperformed the inflammatory, coagulation, and hypoxia-perfusion indices in terms of discrimination (AUC = 0.699). A combined four-dimension model achieved the highest overall discrimination (AUC = 0.744) but provided limited incremental value over electrolyte-metabolic disturbance alone.

**Conclusions:**

Early electrolyte-metabolic disturbance exhibits the strongest and most consistent association with respiratory failure in acute pancreatitis during the first week of hospitalization, underscoring its potential utility for early risk stratification using routinely available laboratory data.

## 1 Introduction

Respiratory failure represents one of the most severe and prognostically significant complications of acute pancreatitis, frequently occurring early in the disease course and accounting for a substantial proportion of short-term mortality (De Troyer et al., 1978; Van Dijk et al., 2017). Despite advances in supportive care, the biological drivers underlying respiratory deterioration remain incompletely defined, and early risk stratification continues to rely largely on composite clinical judgment.

Accumulating evidence suggests that respiratory failure in acute pancreatitis arises from the convergence of multiple pathophysiological processes, including systemic inflammation, coagulation activation, impaired oxygen delivery and tissue perfusion, and electrolyte-metabolic disturbances (De Campos et al., 2007; Singh et al., 2009; Dumnicka et al., 2017; Wu et al., 2024; Xu et al., 2024; Çavdar et al., 2025). Each of these processes has been individually associated with disease severity or adverse outcomes; however, their relative contributions to respiratory failure and their comparative prognostic importance when assessed simultaneously have not been systematically evaluated. Clarifying which biological dimension predominates is clinically relevant, as it may inform prioritization of monitoring, guide early supportive strategies, and refine risk stratification beyond undifferentiated severity labels.

We therefore hypothesized that routinely available laboratory parameters reflecting distinct pathophysiological dimensions would demonstrate unequal associations with respiratory failure and mortality. Specifically, we postulated that electrolyte metabolic disturbance and coagulation activation would exhibit stronger and more independent associations with respiratory failure than inflammatory or hypoxia-perfusion indices when evaluated on a standardized scale. To test this hypothesis, we constructed four composite indices representing systemic inflammation, coagulation, hypoxia-perfusion impairment, and electrolyte-metabolic disturbance, and compared their associative strength, dose-response relationships, and predictive performance in hospitalized patients with acute pancreatitis.

## 2 Methods

### 2.1 Study design and population

**Figure 1:**
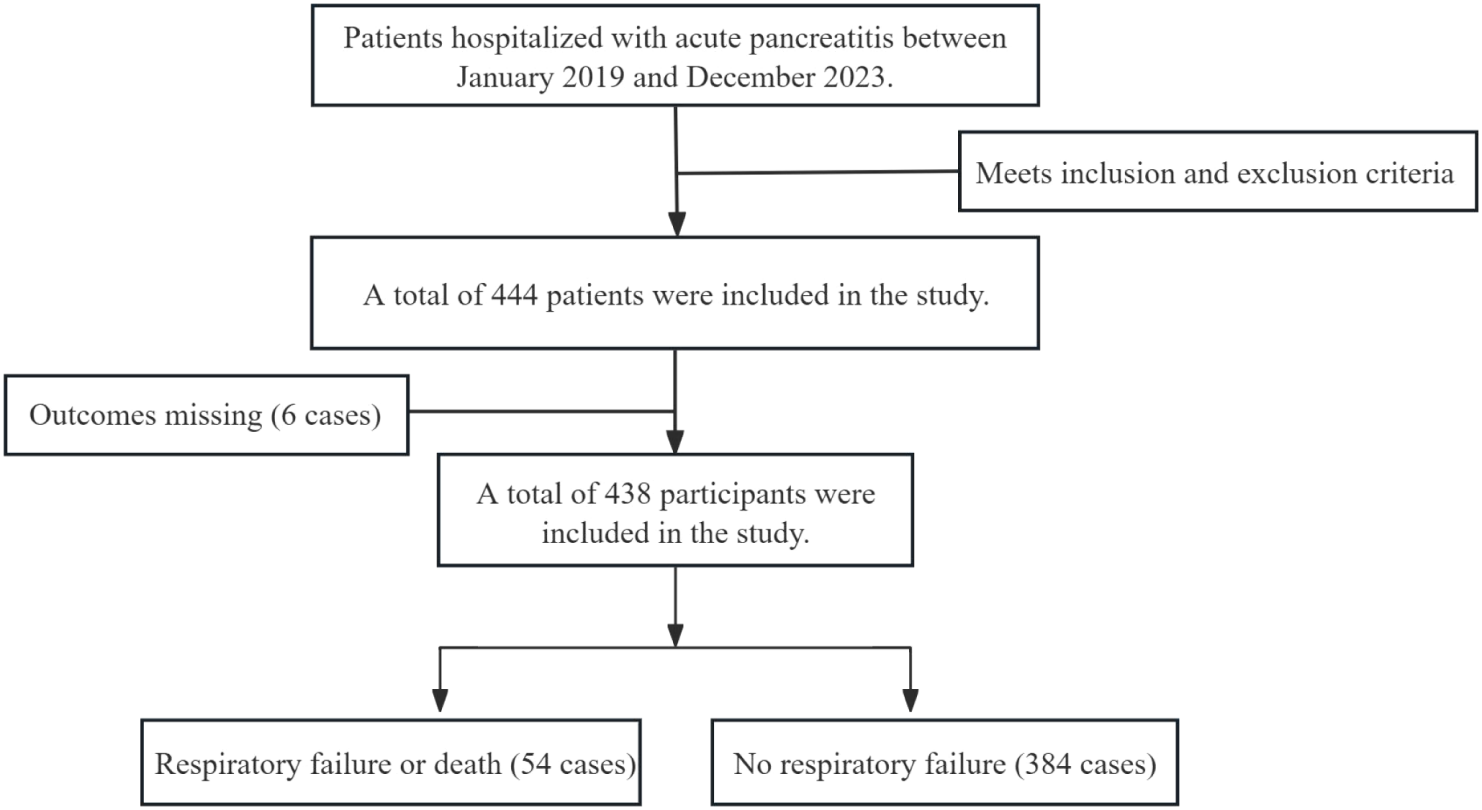
Flowchart of research subject selection.

We conducted a retrospective cohort study involving consecutive adult patients (≥18 years) admitted with acute pancreatitis to a tertiary care center from January 2019 to December 2023. The diagnosis of acute pancreatitis was made based on established international criteria. Patients were deemed eligible if relevant laboratory measurements were completed within 24 hours of admission and the primary outcome could be determined. Out of 1,010 hospitalized patients screened, 444 met the predefined inclusion and exclusion criteria. Six patients were subsequently excluded due to missing outcome data, resulting in a final analysis cohort of 438 patients. Exclusion criteria included pancreatitis secondary to malignancy, pregnancy-associated pancreatitis, death within 24 hours of admission, or excessive missing key clinical data. Among the included patients, 384 were in the no respiratory failure group and 54 in the respiratory failure or respiratory−related death group. The sample size was adequate for multivariable analyses according to established event-per-variable principles. The detailed patient selection process is illustrated in **Figure 1**.

### 2.2 Data collection

Demographic characteristics, comorbidities, and laboratory parameters were extracted from the electronic medical records. All exposure variables were obtained from the first available measurements within 24 hours of admission. These included inflammatory markers (procalcitonin, C-reactive protein, and white blood cell count), coagulation markers (D-dimer), hypoxia and perfusion parameters (hemoglobin, arterial lactate, PaO_2_, PaCO_2_, and FiO_2_), as well as electrolyte and metabolic variables (serum calcium, magnesium, and phosphate).

### 2.3 Composite index construction

Four composite indices were constructed to represent distinct pathophysiological dimensions. To increase the statistical power of the tests, all continuous variables were not converted into categorical variables. To reduce skewness and enhance comparability, variables were log-transformed where appropriate using log(1+x) and standardized to z scores, eliminating the impact of excessively large numerical differences on the model. All indices were scaled such that higher values reflected greater physiological derangement.

Inflammatory index: sum of standardized log-transformed procalcitonin, C-reactive protein, and white blood cell count.

Coagulation index: standardized log-transformed D-dimer.

Hypoxia-perfusion index: standardized combination of hemoglobin, lactate, PaCO_2_, and PaO_2_/FiO_2_.

Electrolyte-metabolic disturbance index: standardized deficits of serum calcium, magnesium, and phosphate relative to the lower limit of normal.

ΔCa = max (0, LLNCa−Ca), LLNCa = 2.02 mmol/L;

ΔMg = max (0, LLNMg−Mg), LLNMg = 0.67 mmol/L;

ΔP = max (0, LLNP−P), LLNP = 0.96 mmol/L.

The hypoxia–perfusion index was designed to reflect disturbances in systemic oxygen delivery and utilization. Hemoglobin represents oxygen-carrying capacity, lactate reflects impaired tissue oxygen utilization and microcirculatory dysfunction, while the PaO_2_/FiO_2_ ratio captures pulmonary gas exchange efficiency. PaCO_2_ was included as an indicator of ventilatory inefficiency and altered alveolar ventilation, which commonly accompanies early respiratory physiological decompensation in acute pancreatitis.

### 2.4 Outcome definition

The primary outcome of this study was defined as early respiratory decompensation occurring within 3 to 7 days after admission. To capture the early phase of respiratory failure that necessitates intensive monitoring or intervention, we utilized a composite outcome. This composite indicator included patients who met the physiological criteria for Type I respiratory failure (defined as a respiratory rate > 30 breaths/min and a partial pressure of arterial oxygen [PaO2] < 60 mmHg on room air or equivalent) (Mirabile et al., 2023), patients who required the initiation of non-invasive or invasive mechanical ventilation, and patients who died from respiratory-related causes during this period. It is important to note that the physiological criteria we selected are intended to identify early respiratory deterioration, a state that typically precedes the formal diagnosis of Acute Respiratory Distress Syndrome (ARDS) as outlined by the Berlin Definition (Ranieri et al., 2012).

### 2.6 Statistical analysis

K-nearest neighbors (KNN) imputation was utilized for continuous variables, while categorical variables with minimal missingness were imputed using the mode. Continuous variables are reported as mean ± standard deviation or median (interquartile range), and categorical variables are presented as counts (percentages). Associations between each composite index and the primary outcome were analyzed using logistic regression, expressed per 1 standard deviation (SD) increase in each index. Models were fitted both unadjusted and adjusted for prespecified covariates, including age, sex, smoking status, alcohol consumption, hypertension, and diabetes. All four indices were entered simultaneously to evaluate their independent associations. Restricted cubic spline models were employed to investigate potential non-linear relationships. The discriminative performance was assessed using the area under the receiver operating characteristic curve (AUC), with comparisons conducted using DeLong’s test. Sensitivity analyses included the exclusion of extreme values and complete-case analyses. All analyses were performed using R version 4.5.2, and two-sided P-values < 0.05 were deemed statistically significant.

To internally validate the construction of our composite indices, a domain-specific Principal Component Analysis (PCA) was conducted. Separate PCAs were performed within the Inflammation, HLO, and EDI domains using their respective standardized biomarkers. The first principal component (PC1) from each domain was extracted to create data-driven, PCA-weighted indices. A sensitivity analysis was then performed by refitting the primary multivariable logistic regression model with these PCA-weighted indices to compare their predictive performance (AUC) and effect sizes (ORs) against the original equal-weight indices.

To address potential overfitting due to an events-per-variable (EPV) ratio of approximately 6, we conducted rigorous internal validation in accordance with the TRIPOD guidelines. First, a Least Absolute Shrinkage and Selection Operator (LASSO) regression with 10-fold cross-validation was performed to verify the robustness of the predictors under penalized conditions. Second, model calibration was assessed using the Brier score and the Hosmer-Lemeshow goodness-of-fit test, accompanied by a calibration plot.

Finally, to address potential residual confounding by baseline disease severity, a sensitivity analysis was conducted by additionally adjusting for the non-respiratory Modified Marshall score and the etiology of pancreatitis (biliary vs. non-biliary).

## 3 Results

### 3.1 Basic characteristics

Compared with patients without respiratory failure, as shown in **Table 1**, those who developed respiratory failure or respiratory-related death exhibited significantly higher levels of the inflammatory index, HLO, EDI, and log1p−transformed D−dimer z score (P<0.001, P = 0.050, P<0.001, and P = 0.001, respectively). Patients with respiratory failure also had a longer length of hospital stay (P = 0.004) and higher organ dysfunction scores, including the Modified Marshall score (P<0.001), SOFA score (P = 0.011), and SIRS score (P<0.001). No significant differences were observed between the two groups with respect to age, sex, smoking status, alcohol use, or comorbidities (all P>0.05).

**Table 1:** Basic characteristic.

Variable	Overall	No respiratory failure	Respiratory failure	t/χ^2^	*P*
Age_z-score	-0.00 ± 1.00	-0.02 ± 0.99	0.17 ± 1.09	-1.21	0.231
Inflammation	-0.00 ± 1.92	-0.11 ± 1.91	0.79 ± 1.77	-3.45	<0.001*
HLO	0.00 ± 1.90	-0.08 ± 1.81	0.60 ± 2.40	-2.00	0.050
EDI	-0.00 ± 1.92	-0.22 ± 1.59	1.57 ± 3.05	-4.24	<0.001*
Log1p_Ddimer_zscore	-0.00 ± 1.00	-0.07 ± 0.96	0.47 ± 1.14	-3.33	0.001*
length of stay	9.98 ± 6.52	9.32 ± 4.65	14.69 ± 12.96	-3.02	0.004*
Modified Marshall score	0.87 ± 0.97	0.79 ± 0.92	1.48 ± 1.11	-4.37	<0.001*
SOFA score	1.76 ± 1.64	1.68 ± 1.61	2.33 ± 1.74	-2.61	0.011*
SIRS criteria score	1.37 ± 1.11	1.26 ± 1.07	2.20 ± 1.05	-6.19	<0.001*
Gender	307 (70.1%)	269 (70.1%)	38 (70.4%)	0.00	1.000
Smoker	244 (55.7%)	210 (54.7%)	34 (63.0%)	1.00	0.317
Alcohol	217 (49.5%)	185 (48.2%)	32 (59.3%)	1.90	0.168
Comorbidity	194 (44.3%)	171 (44.5%)	23 (42.6%)	0.01	0.903

*P < 0.05.

### 3.2 Association analyses

As shown in **Table 2**, in univariable logistic regression models, the inflammatory index, HLO, EDI, and log1p−transformed D−dimer z score were all associated with an increased risk of respiratory failure (odds ratios [ORs] ranging from 1.21 to 1.58; all P ≤ 0.015). These associations remained largely consistent after adjustment for age (z score), sex, smoking status, alcohol use, and comorbidities, with a modest strengthening of the effect estimate for log1p−transformed D−dimer (adjusted OR = 1.63, 95% CI 1.24-2.14; P<0.001). When all indices were simultaneously entered into a single multivariable model (**Table 3**), only EDI remained independently associated with respiratory failure (adjusted OR = 1.32, 95% CI 1.16-1.51; P<0.001), whereas the other indices and covariates did not reach statistical significance.

**Table 2:** Associations of inflammation, HLO, EDI and Log1p_Ddimer_zscore with respiratory failure.

Variable	Univariable logistic regression		Multivariable logistic regression	
	OR (95% CI)	*P*	Adjusted OR (95% CI)	*P*
Inflammation	1.23 (1.08-1.40)	0.002*	1.23 (1.08-1.41)	0.002*
HLO	1.21 (1.04-1.41)	0.015*	1.21 (1.02-1.43)	0.029*
EDI	1.40 (1.24-1.59)	<0.001*	1.40 (1.24-1.59)	<0.001*
Log1p_Ddimer_zscore	1.58 (1.23-2.03)	<0.001*	1.63 (1.24-2.14)	<0.001*

Single-factor logistic regression (unadjusted): Separate logistic regression models were fitted with Inflammation, HLO, EDI, or Log1p_Ddimer_zscore entered individually as the predictor.

Multifactor Logistic Regression (adjusted): For each corresponding Model 1, the model was additionally adjusted for Age_z-score, Gender, Smoker, Alcohol, and Comorbidity.

*P < 0.05.

**Table 3:** Multivariable logistic regression model for respiratory failure.

Variable	Model 1			Model 2		
	B	OR (95% CI)	*P*	B	OR (95% CI)	*P*
Inflammation	0.10	1.11 (0.96-1.28)	0.164	0.11	1.11 (0.96-1.29)	0.163
HLO	0.14	1.15 (0.98-1.34)	0.097	0.13	1.14 (0.96-1.35)	0.149
EDI	0.29	1.33 (1.17-1.52)	<0.001*	0.28	1.32 (1.16-1.51)	<0.001*
Log1p_Ddimer_zscore	0.15	1.16 (0.86-1.56)	0.328	0.18	1.19 (0.87-1.65)	0.279
Age_z-score				0.13	1.14 (0.81-1.61)	0.446
Gender				-0.32	0.72 (0.30-1.78)	0.481
Smoker				0.69	1.99 (0.81-4.88)	0.135
Alcohol				0.22	1.25 (0.60-2.60)	0.557
Comorbidity				-0.22	0.81 (0.43-1.53)	0.506
Constant	-2.18	0.11	<0.001*	-2.40	0.09	<0.001*

A single multivariable logistic regression model was fitted including Inflammation, HLO, EDI, Log1p_Ddimer_zscore, Age_z-score, Gender, Smoker, Alcohol, and Comorbidity simultaneously.

*P < 0.05.

### 3.3 Restricted cubic spline analyses

**Figure 2:**
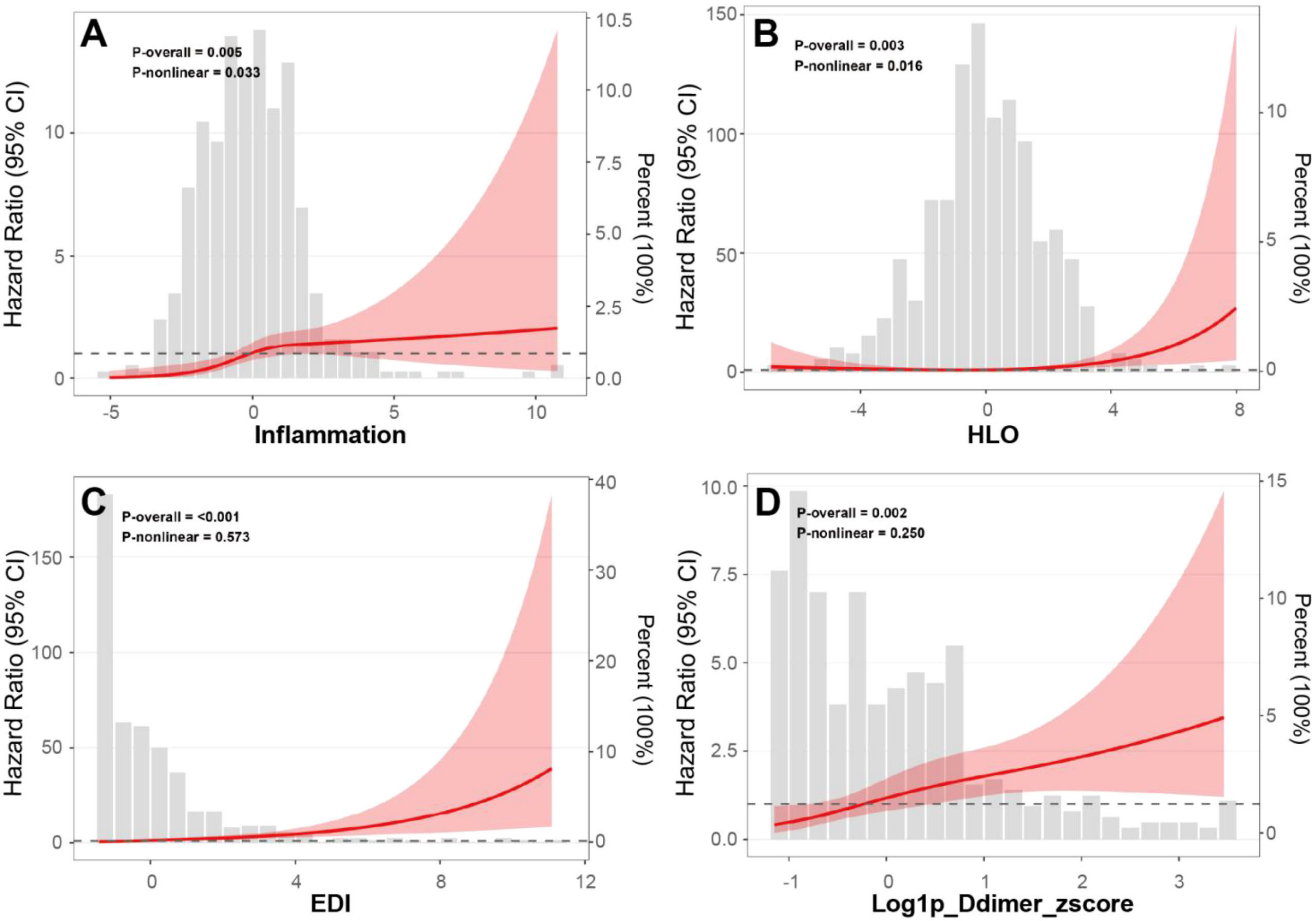
Restricted cubic spline associations of composite indices with respiratory failure. **(A)** Inflammation index; **(B)** Hypoxia-perfusion (HLO) index; **(C)** Electrolyte-metabolic disturbance index (EDI); **(D)** Log1p-transformed D-dimer z-score. Note: The solid lines (red line) represent the estimated odds ratios, and the red shaded areas represent the 95% confidence intervals.

As illustrated in **Figure 2**, restricted cubic spline analyses revealed significant associations between the risk of respiratory failure and the inflammatory index, HLO, EDI, and log1p-transformed D-dimer z score (P for overall association < 0.001, 0.005, 0.002, and 0.003, respectively). Non-linear relationships were identified for the inflammatory index and HLO (P for non-linearity = 0.033 and 0.016, respectively), while no significant non-linearity was observed for EDI or log1p-transformed D-dimer (P for non-linearity = 0.573 and 0.250, respectively).

### 3.4 Discriminative performance

**Figure 3:**
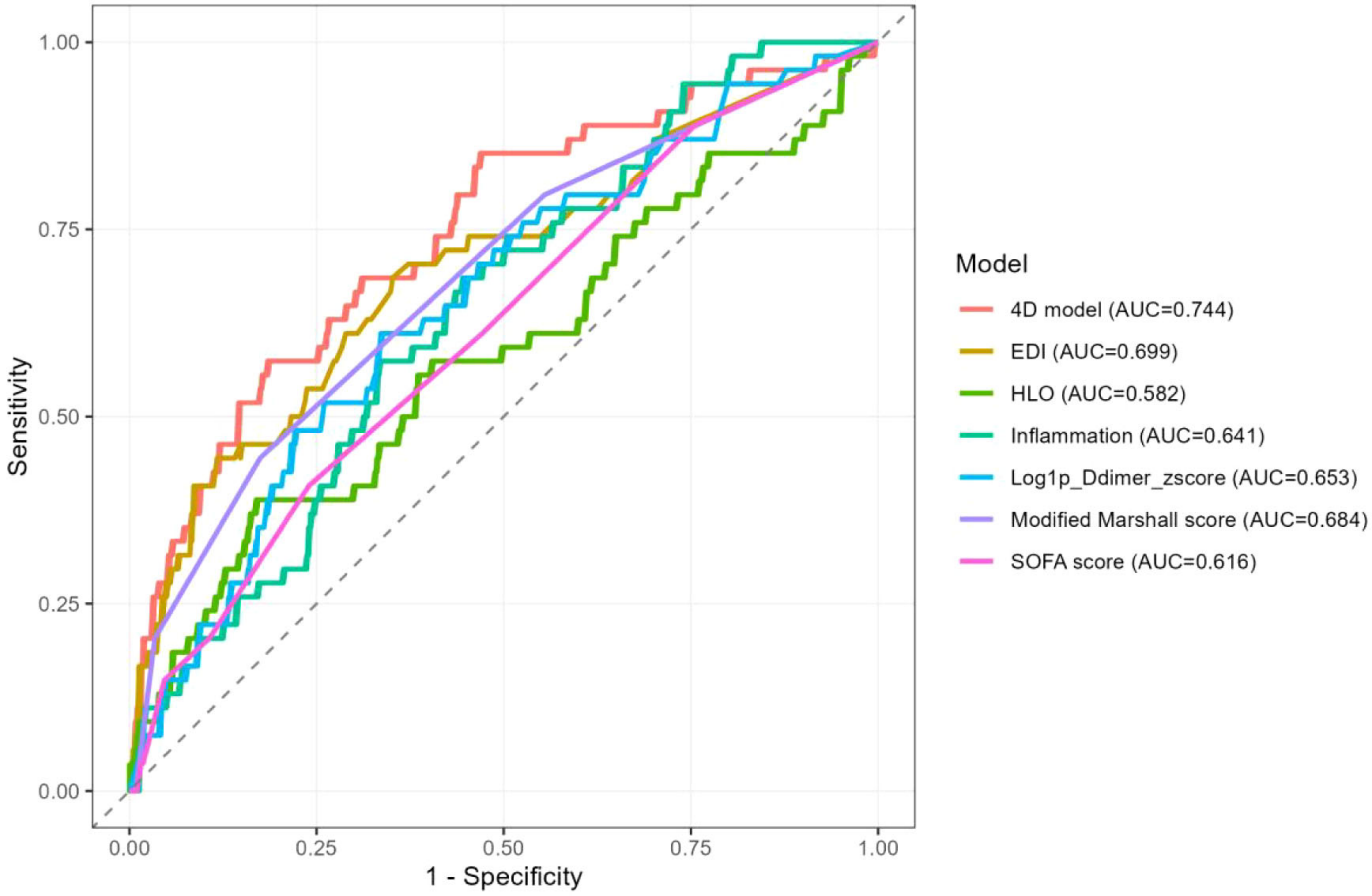
ROC curves for prediction of respiratory failure.

The discriminative performance of the individual indices was modest, with areas under the receiver operating characteristic curve (AUCs) measuring 0.641 for the inflammatory index, 0.582 for HLO, 0.699 for EDI, and 0.653 for the log1p-transformed D-dimer z-score (see **Table 4**). The combined four-dimensional (4D) model exhibited the best overall discrimination, achieving an AUC of 0.744 (95% CI: 0.670-0.819). This model significantly outperformed the inflammatory index, HLO, and log1p-transformed D-dimer (DeLong P-values of 0.005, <0.001, and 0.006, respectively), but did not outperform EDI alone (P = 0.136) (refer to **Table 5**). In comparison with clinical scoring systems, the performance of the Modified Marshall score (AUC 0.684) did not differ significantly from that of the 4D model (P = 0.150), while the SOFA score (AUC 0.616) demonstrated inferior discrimination (P = 0.003). At the optimal cutoff, the 4D model achieved a specificity of 0.815 and an overall accuracy of 0.785 (illustrated in **Figure 3**). To further evaluate clinical utility, we assessed the incremental value of adding EDI to the baseline Modified Marshall score. The addition of EDI significantly improved risk reclassification (Continuous NRI = 0.5845, P < 0.001) and discrimination (IDI = 0.0812, P = 0.001). Decision Curve Analysis (DCA) confirmed that incorporating EDI into the Marshall score provided a higher clinical net benefit across a threshold probability range of 0.05 to 0.30 compared to the Marshall score alone (**Supplementary Figure S1**; **Supplementary Table S1**).

**Table 4:** AUC and DeLong test results for Respiratory failure prediction models.

Model	AUC (95% CI)	p vs 4D model (DeLong)
Inflammation	0.641 (0.568-0.714)	0.005*
HLO	0.582 (0.492-0.672)	<0.001*
EDI	0.699 (0.617-0.781)	0.136
Log1p_Ddimer_zscore	0.653 (0.575-0.732)	0.006*
4D model	0.744 (0.670-0.819)	Reference
Modified Marshall score	0.684 (0.608-0.760)	0.150
SOFA score	0.616 (0.538-0.695)	0.003*

**Table 5:** Diagnostic performance at optimal cut-off values.

Model	Cutoff	Sensitivity	Specificity	PPV	NPV	Accuracy
Inflammation	0.127	0.574	0.667	0.195	0.918	0.655
HLO	0.151	0.389	0.831	0.244	0.906	0.776
EDI	0.102	0.685	0.648	0.215	0.936	0.653
Log1p_Ddimer_zscore	0.123	0.611	0.664	0.204	0.924	0.658
4D model	0.133	0.574	0.815	0.304	0.932	0.785
Modified Marshall score	0.157	0.444	0.826	0.264	0.914	0.779
SOFA score	0.136	0.407	0.760	0.193	0.901	0.717

### 3.5 Internal validation of composite indices

To internally assess the robustness of the prespecified equal-weight indices, domain-specific principal component analysis (PCA) was performed (**Supplementary Figures S2**, **S3**; **Supplementary Table S2**). The PCA-derived scores for the Inflammation and EDI domains showed very high correlations with the corresponding equal-weight indices (r=0.970and r=0.987, respectively). When the primary multivariable model was refitted using the PCA-derived scores, model discrimination was essentially unchanged compared with the original equal-weight model (AUC: 0.748 vs. 0.741). In addition, the direction and statistical significance of the associations remained consistent, with the EDI continuing to show the strongest association with the primary outcome. As data-driven weighting provided no meaningful improvement in predictive performance, the equal-weight indices were retained in the primary analysis for parsimony and clinical interpretability.

### 3.6 Internal validation and model calibration

Given the EPV of 6, internal validation was performed to assess model stability. In the LASSO penalized regression, the EDI remained the strongest retained predictor (coefficient = 0.278), confirming its robust independent association with the outcome despite the L1 penalty. Furthermore, the model demonstrated adequate calibration, with a Brier score of 0.095 and a non-significant Hosmer-Lemeshow test (P = 0.204). A comprehensive overview of the validation metrics, including the calibration plot and LASSO regularization path, is provided in **Supplementary Figure S4** and **Supplementary Table S3**.

### 3.7 Sensitivity analyses

**Figure 4:**
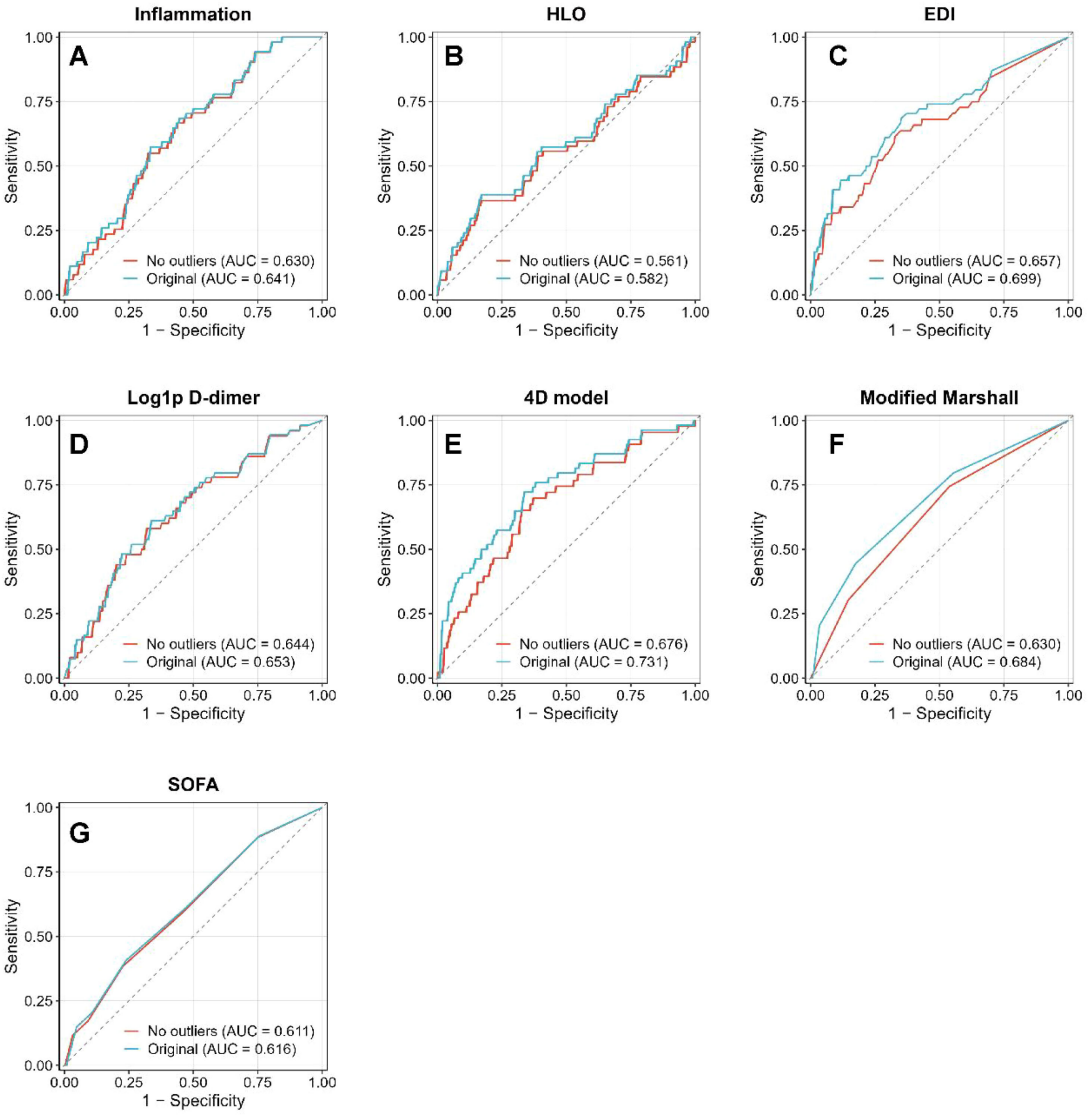
Sensitivity analyses of discriminative performance after exclusion of outliers. Receiver operating characteristic (ROC) curves comparing the original models (blue lines) with models excluding outliers (red lines) for: **(A)** Inflammation index; **(B)** Hypoxia-perfusion (HLO) index; **(C)** Electrolyte-metabolic disturbance index (EDI); **(D)** Log1p-transformed D-dimer z-score; **(E)** Combined four-dimensional (4D) model; **(F)** Modified Marshall score; and **(G)** SOFA score.

As shown in **Figure 4**, after exclusion of outliers, the discriminative performance of all models remained broadly consistent with the primary analysis, with only minor changes in AUC values: the inflammatory index decreased from 0.641 to 0.630, HLO from 0.582 to 0.561, EDI from 0.699 to 0.657, and log1p−transformed D−dimer z score from 0.653 to 0.644; the 4D model decreased from 0.731 to 0.676. Clinical scores also remained stable (Modified Marshall score from 0.684 to 0.630; SOFA score from 0.616 to 0.611), indicating that the findings were robust to the handling of outliers.

To account for potential confounding by baseline disease severity, we performed an additional sensitivity analysis adjusting for the non-respiratory Modified Marshall score and pancreatitis etiology. As shown in **Supplementary Figure S5**, the independent association between EDI and respiratory failure remained robust (Adjusted OR = 1.32, 95% CI: 1.15-1.51, P < 0.001), indicating that early electrolyte-metabolic disturbance is not merely a secondary epiphenomenon of overall severity but an independent predictor.

## 4 Discussion

In this retrospective cohort study, we compared the relative associative and predictive value of four distinct pathophysiological dimensions for early respiratory failure or death in acute pancreatitis. Our principal finding is that electrolyte-metabolic disturbance, summarized by the EDI, demonstrated the most robust and independent association with early adverse respiratory outcomes, outperforming inflammatory, coagulation, and hypoxia-perfusion indices when evaluated simultaneously. These findings extend prior evidence that early organ failure, particularly respiratory failure, is a major determinant of short−term prognosis in acute pancreatitis (Boxhoorn et al., 2020; Mederos et al., 2021), and suggest that metabolic derangement may play a more central role in early respiratory deterioration than is currently emphasized.

Systemic inflammation and coagulation activation are well −established contributors to organ failure in acute pancreatitis and were each associated with respiratory failure in univariable and partially adjusted models. Experimental and clinical studies have shown that cytokine−mediated endothelial injury and activation of coagulation cascades contribute to pulmonary microvascular dysfunction and acute lung injury in this setting (Schmandt et al., 2021; Zhang et al., 2021; Liu et al., 2024). However, the attenuation of these associations in fully adjusted models suggests substantial overlap among inflammatory, thrombotic, and perfusion−related pathways, supporting the concept that early respiratory failure reflects a convergence of interrelated pathophysiological processes rather than the dominance of a single axis (Mederos et al., 2021; Trikudanathan et al., 2024).

In contrast, electrolyte-metabolic disturbance remained independently associated with respiratory failure across all modeling strategies and exhibited a near−linear dose-response relationship. Hypocalcemia, hypomagnesemia, and hypophosphatemia are common in acute pancreatitis and have been associated with disease severity and adverse outcomes in contemporary cohorts (Fischman et al., 2023; Fernandes and Pereira, 2024; Li et al., 2024; Pergolini et al., 2024). These abnormalities may directly impair diaphragmatic contractility, neuromuscular transmission, and cellular energy metabolism, thereby predisposing patients to early ventilatory failure (Zerem et al., 2023; Song and Lee, 2024). Beyond their mechanistic relevance, electrolyte disturbances may also function as integrative biomarkers, capturing downstream effects of systemic inflammation, microcirculatory dysfunction, and mitochondrial stress (Bos and Ware, 2022; Fernandes and Pereira, 2024). Our findings therefore suggest that electrolyte-metabolic derangement is not merely a secondary epiphenomenon, but a clinically meaningful indicator of early physiological decompensation.

From a risk−stratification perspective, the combined four−dimension (4D) model demonstrated good discriminatory performance and compared favorably with established clinical scoring systems. Nevertheless, its limited incremental value over EDI alone highlights the practical appeal of parsimonious, mechanism−informed indices derived from routinely available laboratory data. This observation is consistent with recent emphasis on early simplicity, interpretability, and clinical applicability in acute pancreatitis risk assessment (Boxhoorn et al., 2020; Beij et al., 2025).

Several limitations warrant consideration. First and foremost, the retrospective, single-center design restricts causal inference and limits the generalizability of our findings to other populations or healthcare settings. There is an urgent need for prospective, multicenter external validation before these indices can be reliably applied in routine clinical practice. Second, laboratory measurements were confined to the first 24 hours of admission, thereby precluding the assessment of temporal trajectories that may further refine risk prediction. Third, despite multivariable adjustment and sensitivity analyses, residual confounding cannot be entirely excluded. Finally, although the composite indices were constructed *a priori* to reflect distinct biological domains, alternative biomarker selections or weighting strategies may yield different results.

In conclusion, our study demonstrates that early electrolyte-metabolic disturbances exhibit the strongest and most independent association with respiratory failure or death in acute pancreatitis among multiple competing physiological dimensions. These findings underscore the potential clinical significance of early metabolic monitoring and suggest that electrolyte-focused indices may complement or refine existing approaches to early risk stratification. Prospective multicenter studies are warranted to validate these observations and to determine whether early correction of electrolyte-metabolic abnormalities can modify respiratory outcomes.

## 5 Conclusion

In this study, we systematically compared four pathophysiological dimensions associated with early respiratory failure or respiratory-related death in acute pancreatitis. Among these dimensions, electrolyte-metabolic disturbance exhibited the strongest and most consistent association with early respiratory failure or mortality, independent of inflammatory, coagulation, and hypoxia-perfusion abnormalities. These findings underscore the clinical significance of early metabolic assessment and suggest that electrolyte-focused indices derived from routine laboratory data may improve early risk stratification in acute pancreatitis.

## Data Availability

The de-identified clinical dataset used in this study has been openly shared on Science Data Bank (ScienceDB). The dataset includes demographic characteristics, comorbidities, and raw laboratory measurements (inflammatory markers, coagulation profiles, blood gas analyses, and electrolyte panels) obtained within 24 hours of admission. Data preprocessing steps, including K-nearest neighbors (KNN) imputation for missing values, log(1+x) transformations for skewed continuous variables, and z-score standardization prior to composite index construction, are documented alongside the dataset. The dataset can be accessed via the following persistent identifier: DOI: 10.57760/sciencedb.34037.
