# Supplementary material for: Comparative associative and discriminative value of inflammatory, coagulation, hypoxia–perfusion, and electrolyte–metabolic indices for early respiratory physiological decompensation in acute pancreatitis
Source: Front Physiol. 2026 Apr 22;17:1775368. doi: 10.3389/fphys.2026.1775368 (PMC13143626; doi:10.3389/fphys.2026.1775368)

**Figure S1. Decision curve analysis of Marshall Score, EDI, Marshall + EDI, and 4D model**


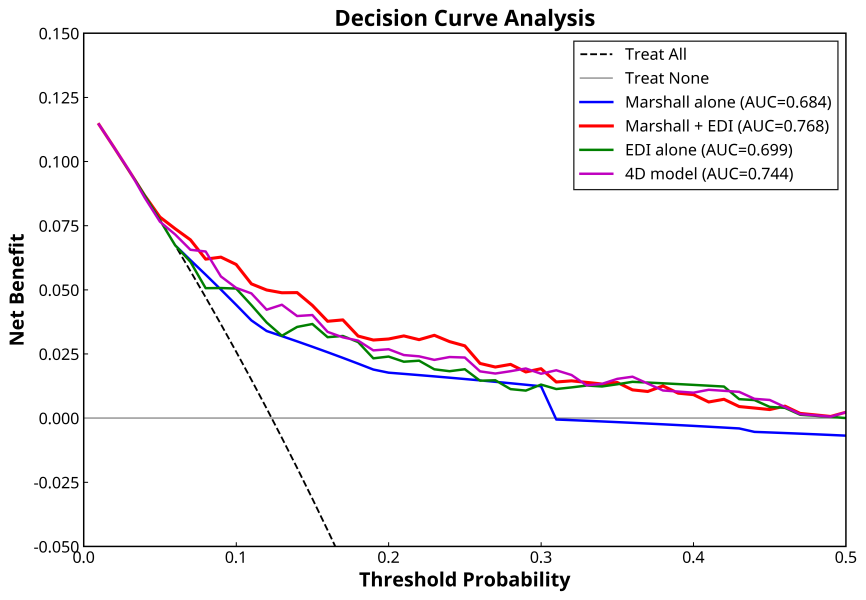


**Table S1. Incremental predictive value of EDI and the 4D model beyond Marshall score**

| **Comparison** | **Base AUC** | **New AUC** | **Delta AUC** | **Continuous NRI** | **NRI P-value** | **IDI** | **IDI P-value** |
| --- | --- | --- | --- | --- | --- | --- | --- |
| Marshall+EDI vs Marshall | 0.684 | 0.768 | 0.084 | 0.584 | 0.000 | 0.081 | 0.001* |
| 4D model vs Marshall | 0.684 | 0.744 | 0.060 | 0.287 | 0.048 | 0.063 | 0.015* |

**Figure S2. Forest plot of odds ratios for equal-weight and PCA-weight composite indices**


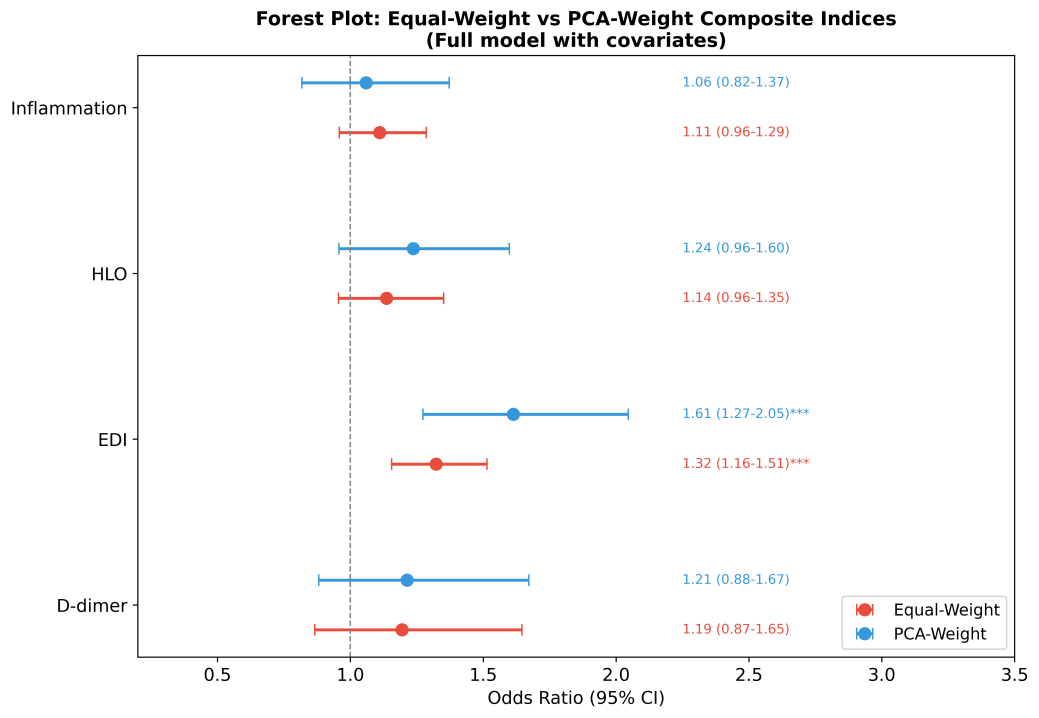


**Figure S3. ROC curve comparison of equal-weight and PCA-weight models for early respiratory decompensation**


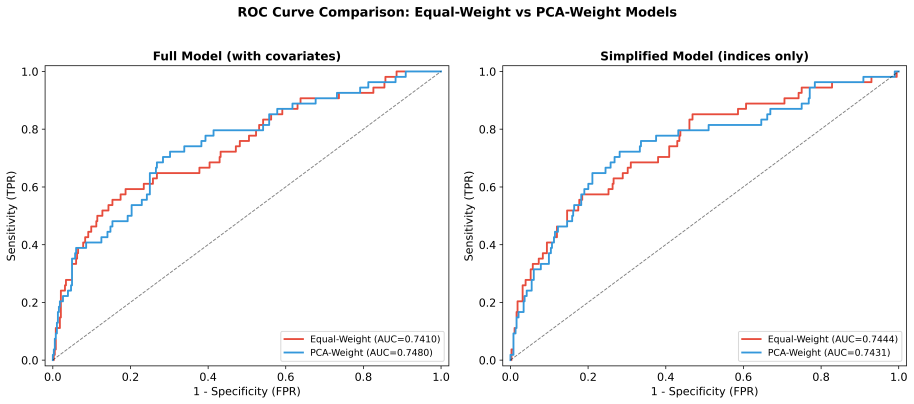


**Table S2. Domain-specific PCA results for composite indices**

| **Domain** | **Variable** | **PC1 Loading** | **PC1 Variance Explained %** | **KMO** | **Bartlett Chi2** | **Bartlett P** |
| --- | --- | --- | --- | --- | --- | --- |
| Inflammation | PCT | 0.639 | 41.573 | 0.506 | 25.293 | <0.001* |
| Inflammation | CRP | 0.694 | 41.573 | 0.506 | 25.293 | <0.001* |
| Inflammation | WBC | 0.331 | 41.573 | 0.506 | 25.293 | <0.001* |
| HLO | Hemoglobin | 0.390 | 33.450 | 0.482 | 50.799 | <0.001* |
| HLO | Lactate | 0.699 | 33.450 | 0.482 | 50.799 | <0.001* |
| HLO | PaO2/FiO2 | -0.293 | 33.450 | 0.482 | 50.799 | <0.001* |
| HLO | PaCO2 | -0.522 | 33.450 | 0.482 | 50.799 | <0.001* |
| EDI | ΔCalcium | 0.667 | 41.485 | 0.525 | 21.896 | <0.001* |
| EDI | ΔMagnesium | 0.416 | 41.485 | 0.525 | 21.896 | <0.001* |
| EDI | ΔPhosphorus | 0.618 | 41.485 | 0.525 | 21.896 | <0.001* |

**Figure S4. Internal validation panel: LASSO path, coefficient comparison, and calibration plot**


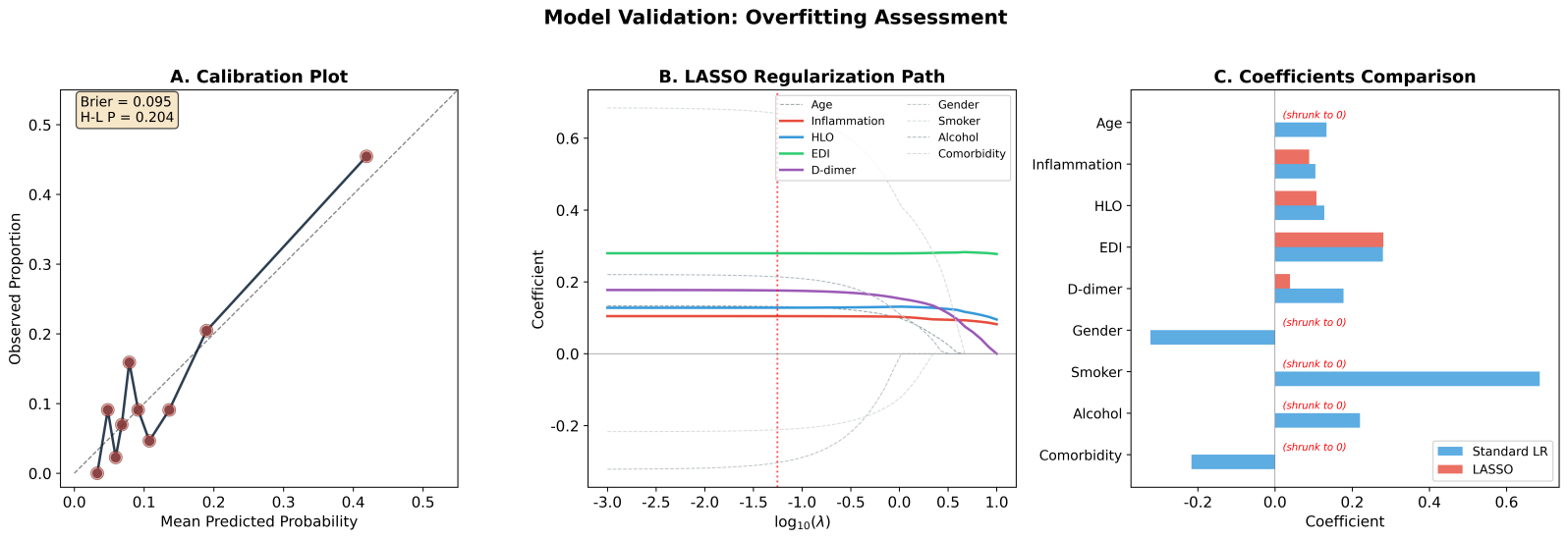


**Table S3. Penalized and standard logistic regression results**

| **Variable** | **LASSO Coefficient** | **Retained** | **Standard LR Coefficient** | **Shrinkage Pct** |
| --- | --- | --- | --- | --- |
| age zscore | 0 | FALSE | 0.133 | 100 |
| Inflammation index | 0.089 | TRUE | 0.105 | 15.320 |
| HLO index | 0.107 | TRUE | 0.128 | 16.041 |
| EDI index | 0.281 | TRUE | 0.280 | -0.419 |
| Ddimer zscore | 0.039 | TRUE | 0.177 | 78.282 |
| gender | 0 | FALSE | -0.323 | 100 |
| smoker | 0 | FALSE | 0.686 | 100 |
| alcohol | 0 | FALSE | 0.220 | 100 |
| comorbidity | 0 | FALSE | -0.217 | 100 |

**Figure S5. Sensitivity analysis forest plot of EDI and early respiratory decompensation**


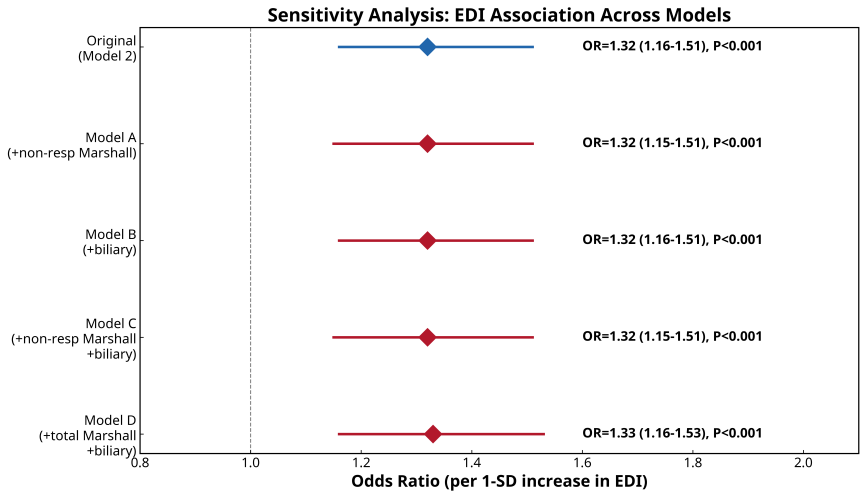

Supplement: Supplementary file 1 [file SupplementaryFile1.docx]
